# Detection and molecular characterization of the novel recombinant norovirus GII.P16-GII.4 Sydney in southeastern Brazil in 2016

**DOI:** 10.1371/journal.pone.0189504

**Published:** 2017-12-13

**Authors:** Débora Maria Pires Gonçalves Barreira, Túlio Machado Fumian, Marco André Loureiro Tonini, Lays Paula Bondi Volpini, Rodrigo Pratte Santos, Anézia Lima Chaves Ribeiro, José Paulo Gagliardi Leite, Márcia Terezinha Baroni de Moraes e Souza, Patrícia Brasil, Denise Cotrim da Cunha, Marize Pereira Miagostovich, Liliana Cruz Spano

**Affiliations:** 1 Laboratory of Virology and Infectious Gastroenteritis, Pathology Department, Health Science Center, Federal University of Espírito Santo, Av. Marechal Campos 1468, Maruípe, Vitória, ES, Brazil; 2 Laboratory of Comparative and Environmental Virology, Oswaldo Cruz Institute, Av. Brasil 4365, Rio de Janeiro, RJ, Brazil; 3 Central Laboratory of Public Health, Av. Marechal Mascarenhas de Moraes 2025, Bento Ferreira, Vitória, ES, Brazil; 4 Acute Febrile Illnesses Laboratory, Evandro Chagas National Institute of Infectious Diseases, Av. Brasil 4365, Rio de Janeiro, RJ, Brazil; 5 Sérgio Arouca Public Heath National School, Av. Brasil 4365, Rio de Janeiro, RJ, Brazil; CEA, FRANCE

## Abstract

Noroviruses are the leading cause of acute gastroenteritis (AGE) in all age groups worldwide. Despite the high genetic diversity of noroviruses, most AGE outbreaks are caused by a single norovirus genotype: GII.4. Since 1995, several different variants of norovirus GII.4 have been associated with pandemics, with each variant circulating for 3 to 8 years. The Sydney_2012 variant was first reported in Australia and then in other countries. A new variant, GII.P16-GII.4, was recently described in Japan and South Korea and then in the USA, France, Germany and England. In our study, 190 faecal specimens were collected from children admitted to a paediatric hospital and a public health facility during a surveillance study of sporadic cases of AGE conducted between January 2015 and July 2016. The norovirus was detected by RT-qPCR in 51 samples (26.8%), and in 37 of them (72.5%), the ORF1-2 junction was successfully sequenced. The new recombinant GII.P16-GII.4 Sydney was revealed for the first time in Brazil in 2016 and predominated among other strains (9 GII.Pe-GII.4, 3 GII.P17-GII.17, 1 GII.Pg-GII.1, 1 GII.P16-GII.3 and 1 GII.PNA-GII.4). The epidemiological significance of this new recombinant is still unknown, but continuous surveillance studies may evaluate its impact on the population, its potential to replace the first recombinant GII.Pe-GII.4 Sydney 2012 variant, and the emergence of new recombinant forms of GII.P16.

## Introduction

Noroviruses, belonging to the genus *Norovirus* in the *Caliciviridae* family, are a leading cause of acute gastroenteritis (AGE) in all age groups worldwide [1]. Annually, it is estimated that noroviruses account for more than 200,000 deaths globally, with significant disease burdens in developing countries [2]. Noroviruses can be subdivided into seven genogroups (G), of which GI, GII and GIV have been detected in humans, and they can be further divided into 33 genotypes [3]. The norovirus-positive single-stranded RNA genome is organized into three open reading frames (ORFs 1, 2 and 3): ORF1 encodes non-structural proteins, ORF2 encodes the major structural protein (VP1), and ORF3 encodes the minor structural protein (VP2) [4]. The VP1 protein is organized into the N-terminal (N), the shell (S), and the protruding (P) domains, and the last domain can be further divided into two subdomains, P1 and P2. The P2 subdomain is exposed on the surface of the capsid protein and contains the major antibody recognition epitopes (A-E) and the histo-blood group antigen (HBGA) binding domains [5, 6].

These viruses are genetically diverse, with genetic drift and recombination being the main driving forces shaping their evolution [7]. It is well known that the norovirus recombination hotspot is located near the ORF1/ORF2 overlapping region, and a variety of recombinant strains have been reported in Brazil and elsewhere [8, 9, 10, 11]. Despite its high genetic diversity, most AGE outbreaks are caused by a single norovirus genotype: GII.4 [1, 12]. Since 1995, several different variants of GII.4 have emerged, remaining in human populations for 3 to 8 years, and they are associated with the global increase in AGE outbreaks [13]. The current pandemic variant (GII.4 Sydney), first detected in Australia in 2012, originated from an antigenic capsid variation and recombination event (GII.Pe-GII.4), and it rapidly spread globally [14].

A novel norovirus recombinant strain, GII.P16-GII.4, emerged in 2015 in the USA, and it has been the predominant outbreak strain since then [15]. In September 2016, this strain was described as the cause of an AGE outbreak in patients in Kawasaki City, Japan [16]. Since then, GII.P16-GII.4 has been reported in sporadic cases and outbreaks from September to December 2016 in Germany and in increased outbreaks during 2016/2017 in France, and it was detected in the waters of coastal streams in South Korea in December 2015, replacing the formerly dominant strain GII.P17-GII.17 [17, 18]. The last report came from sporadic cases and outbreaks during 2015–2016 in England [19]. Our study documents for the first time the emergence of this strain in samples from children with AGE on the southeastern coast of Brazil.

## Materials and methods

### Specimens and ethics statement

Faecal specimens were collected from 190 children up to 11 years old during a surveillance study of sporadic cases of AGE conducted between January 2015 and July 2016 in the southeastern Brazilian coast state, Espírito Santo. Inpatients and outpatients were enrolled at a paediatric hospital and a public health facility located in the city of Vitória (state capital) to assess norovirus and rotavirus A frequencies. Three additional norovirus-positive specimens were included in the study. They were obtained from an ongoing community-based project that aims to monitor the incidence of norovirus and rotavirus A from asymptomatic and symptomatic children living in Manguinhos, a shantytown in northern Rio de Janeiro state, also on the southeastern Brazilian coast. All specimens were obtained after parents signed the consent form. The studies were approved by either the Institutional Ethical Research Committee of the Center of Health Sciences of the Federal University of Espírito Santo (No. 704.923/14) or the Oswaldo Cruz Foundation–CEP/ENSP/Fiocruz n° 688.566/14).

### RNA extraction and norovirus detection

Viral RNA was extracted from 10% faecal suspensions (w/v), and the nucleic acids were extracted using the commercial QIAamp^®^ Viral RNA Mini Kit (Qiagen, Valencia, CA, USA) according to the manufacturer's instructions. Initially, norovirus detection was performed by RT-qPCR using primers and a probe targeting the ORF1/2 junction [10, 20].

### Molecular characterization, phylogenetic and recombination analysis

For norovirus characterization, positive samples were amplified by using the One-Step RT-PCR kit (QIAGEN, Valencia, CA, USA) with Mon431 (nt 4820–4839) and G2SKR (nt 5367–5389) primers targeting a 570 bp fragment covering the 3’-end ORF1 and 5’-end ORF2, named regions B and C, respectively [21, 22]. Sequencing was performed using the BigDye^®^ Terminator v3.1 Cycle Sequencing Kit and ABI Prism 3730 Genetic Analyser (Applied Biosystems, Foster City, CA, USA), and the genotype was assigned using the Norovirus Automated Genotyping Tool [23]. Phylogenetic trees (based on the partial sequences of ORFs1 and 2) were constructed using the neighbour-joining method (Kimura two-parameter model, 2000 bootstrap replications for branch support) in MEGA 6.0 [24].

Plot similarity was carried out using SimPlot version 3.5.1 [25] to confirm the recombination event and to identify a putative recombination breakpoint. SimPlot analysis was performed by setting the window width and the step size to 200 bp and 20 bp, respectively. In addition, different methods implemented in the Recombination Detection Programme v.4.16 (RDP4) were also used [26], such as Bootscan, Recscan, GENECONV, Maxchi, and Chimaera analysis. One of the recombinant strains obtained in our study (LVCA_25726) was included as a query, while putative parental sequences were obtained from the GenBank database. Events detected by RDP with a p-value less than 10^−5^ were considered significant. The 40 sequences of the ORF1/2 region were deposited in GenBank under the accession numbers KY451971-KY451987 and MF158177-MF158199.

### P2 region analysis

The P2 subdomains of three samples were amplified using the primers EVP2F and EVP2R [27] to explore nucleotide and amino acid changes in the major epitopes of the VP1 protein. The four P2 sequences were deposited in GenBank under the accession numbers MF681695-MF681696 and KY551568-KY551569.

## Results

**Fig 1:**
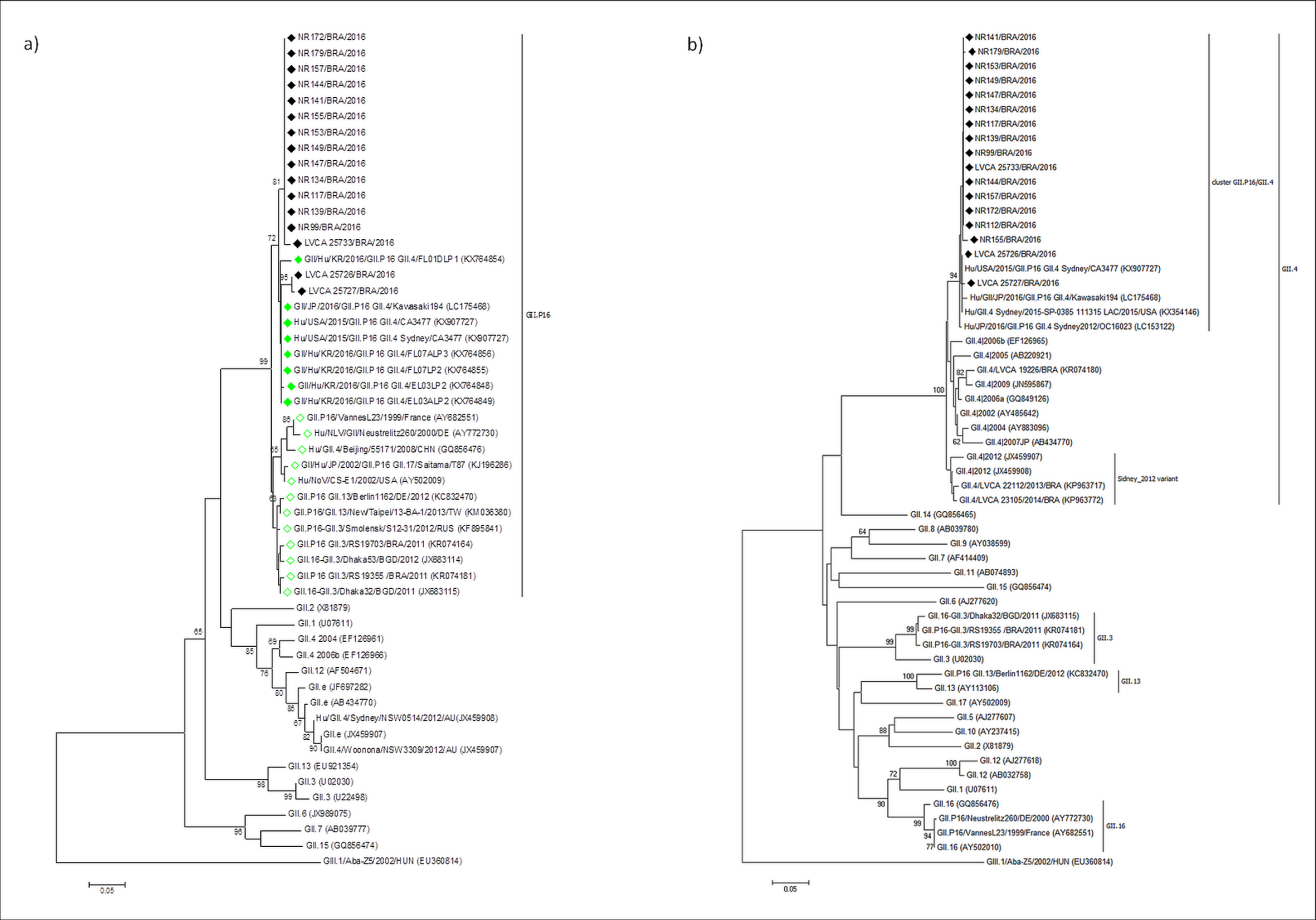
Phylogenetic analysis of GII norovirus based on the partial nucleotide sequences of the polymerase and capsid regions, using the MON431 (nt 4820–4839) and G2SKR (nt 5367–5389) primers. (a) Phylogenetic tree of 231 bp within the polymerase region (3’-ORF1). (b) Phylogenetic tree of 277 bp within the capsid region (5’-ORF2). References strains of norovirus genotypes are named according to GenBank with their respectively accession numbers. Brazilian strains are marked with black filled diamonds. Recombinant strains of GII.P16-GII.4 are marked with green filled diamonds, and recombinant strains of GII.P16 grouping with non-GII.4 capsid genotypes are highlighted with unfilled green diamonds. The bootstrap values (2,000 replicates) are indicated in the phylogenetic tree, and values less than 70% are not represented. The bar at the bottom of the figure is proportional to the genetic distance.

A total of 26.8% (51/190) of the samples were positive for the GII norovirus, all in children up to four years old, and no GI genotypes were detected; 37 strains from among the 51 noroviruses were successfully sequenced. Based on results obtained with the genotyping tool (http://www.rivm.nl/mpf/norovirus/typingtool) and from both the polymerase and capsid phylogenetic trees, most (59.5%) of the strains characterized (22/37) were classified as the novel norovirus recombinant form GII.P16-GII.4 Sydney (Table 1, Fig 1), including the three strains from Rio de Janeiro, followed by GII.Pe-GII4 Sydney 2012 (24.3%). The two strains were not co-circulating, and since the first detection of the newly emergent GII.P16-GII.4, it has predominated among the strains characterized (Table 1).

**Table 1:** Distribution of norovirus genotypes detected in samples collected between January 2015 and July 2016 (n = 37) of the southeastern Brazilian coast state, Espírito Santo.

YEAR	Norovirus sequenced region ORF-1 ORF-2		Number of strains (%) N = 37
2015	GII.P16	GII.3	1 (2.7)
	GII.Pg	GII.1	1 (2.7)
	GII.Pe	GII.4 Sydney 2012	9 (24.3)
2016	GII.PNA	GII.4	1 (2.7)
	GII.P17	GII.17	3 (8.1)
	GII.P16	GII.4 Sydney	22 (59.5)

All the samples characterized as the new recombinant strain containing the GII.P16 polymerase genotype were detected between February and June 2016, and they represented 84.6% (22/26) of all genotyped samples in that period. Three samples were characterized as GII.P17-GII.17, a formerly emergent genotype, in the months of January to May 2016.

The 22 GII.P16 sequences detected in our study belong to the recent emergent recombinant form grouped into a separate clade containing GII.P16-GII.4 Sydney strains that were recently detected in the USA (KX907727) and Japan (LC175468) in the years 2015 and 2016, respectively (Fig 1A). The other detected RdRp sequence of GII.P16 present in a different recombinant type (GII.3 capsid genotype) was grouped into another phylogenetic clade along with strains detected before 2014, represented by recombinant forms with different capsid genotypes, such as GII.13 and GII.3 (Fig 1B).

**Fig 2:**
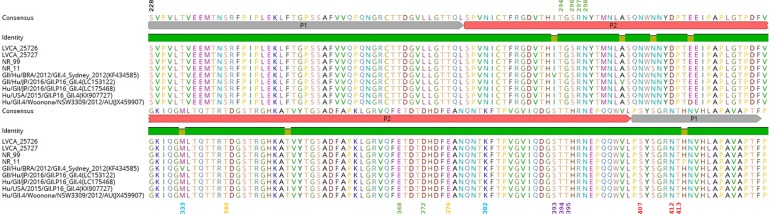
Alignment of sequences of VP1 protruding P domain derived from norovirus GII.4 strains. Antigenic epitopes A (green), B (blue), C (orange), D (purple) and E (red) are indicated.

SimPlot analysis confirmed the recombination event between GII.P16 and GII.4 Sydney and revealed the breakpoint location near the ORF1-2 junction for all strains (data not shown), like the strains described in Japan [15]. When comparing four strains with the reference strain Sydney 2012 (JX459907), other than an exchange of an amino acid (aa) at position 315 (all strains) and at positions 309 and 414 (NR11), which are outside the main antigenic sites (epitopes A-E), we observed no other aa changes (Fig 2).

## Discussion

In Brazil, a great diversity of norovirus GII.4 variants, as well as intra-genotype recombinant strains, have circulated throughout the country, following the global pattern observed in many studies [28, 29, 30]. Herein, we described the detection in Brazil of an emergent recombinant genotype that is currently circulating in many countries worldwide [15, 16, 17, 18, 19]. During the study period, we observed a change in the circulation pattern of the recombinant strains, with GII.Pe-GII.4 Sydney 2012 predominant in 2015, followed by an emergence and predominance of GII.P16-GII.4 Sydney in 2016.

The emergence and predominance of the novel recombinant GII.P16-GII.4 Sydney in 2016 in southeastern Brazil corroborates with a similar trend in the current genotype circulation observed in other countries, such as the USA, Japan, France, England and South Korea [15, 16, 17, 19, 31]. In the USA, GII.P16-GII.4 Sydney predominated in the winter of 2015–2016 and became responsible for 60% of the total norovirus AGE outbreaks during the winter of 2016–2017 (https://www.cdc.gov/norovirus/reporting/caliciNet/data-tables.html) [15].

During this study, we also detected the formerly emergent GII.P17-GII.17 in three samples analysed from 2016. This emergent virus had already been detected in Brazil in 2015 [32, 33]; however, despite a global distribution, its major impact in causing AGE outbreaks was observed mainly in Asian countries [34, 35, 36, 37].

It is noteworthy that despite GII.4 being the most prevalent genotype worldwide, limited types of polymerase recombination are described, such as GII.P1, GII.P12, GII.Pe and GII.P21 [9, 38, 39], suggesting that there is a restriction mediated by the efficiency of viral replication [38]. In turn, the GII.P16 polymerase, albeit less common, has already been detected as being associated with the GII.3 capsid genotype in Spain, Bangladesh and Italy [40, 41, 42], with GII.2 in China and Japan [43, 44], GII.13 in Italy and Nepal [42, 45] and GII.17 in South Africa [46]. In our study, GII.P16 was detected with the GII.3 capsid genotype circulating in 2015; however, it was grouped in a different cluster than our GII.P16-GII.4 Sydney strains and previous strains detected in AGE outbreaks in Southern Brazil between 2010 and 2011 [10]. Recently, GII.P16 re-emerged combined with GII.2 grouped in the same cluster as the polymerase GII.P16 of the new recombinant GII.P16-GII.4 Sydney, differing from the previous one and suggesting the importance of the current GII.P16 polymerase in norovirus circulation worldwide [18, 19, 47, 48, 49, 50, 51, 52]. The authors also described the importance of the polymerase for viral fitness and variability [19]; therefore, the newly acquired polymerase could play a role in the success of this recombinant form.

Although aa changes in major epitopes are important to norovirus evolution, especially epitopes A and D, which played a role in the emergence of the last variant GII.4 Sydney 2012 [53], the three strains analysed in our study showed no aa changes in any of these sites compared with the GII.4 Sydney 2012 reference strain (JX459907), or with GII.P16-GII.4 strains detected in Japan and the USA (Fig 2). Similar results were obtained by Ruis et al. (2017), who reported no amino acid substitutions in the capsid of GII.P16 strains isolated in England between 2015 and 2016, indicating the inability of this new recombinant strain to escape immunity against the old GII.Pe-GII.4 Sydney 2012.

In conclusion, we described the newly emergent GII.P16-GII.4 Sydney strain circulating along the southeastern coast of Brazil and becoming the predominant strain among samples from this region analysed in 2016, having replaced the formerly emergent genotype GII.Pe-GII.4 Sydney. The epidemiological impact of this emergent recombinant strain is still unknown; however, it has already been detected circulating in several countries, suggesting a potential worldwide distribution.

The genetic novelty of the new recombinant strains could reside in the acquisition of a more efficient polymerase [19, 52]. The sequencing of both polymerase and capsid genes is a valuable tool for a proper understanding of norovirus epidemiology and evolution. Concerning this new variant, future studies are needed to investigate how long this recombinant will remain circulating and whether new forms of GII.P16 recombination will emerge.
